# Flavonoids from *Rosa* *roxburghii *Tratt prevent reactive oxygen species-mediated DNA damage in thymus cells both combined with and without PARP-1 expression after exposure to radiation *in vivo*

**DOI:** 10.18632/aging.103688

**Published:** 2020-08-29

**Authors:** Sai-Juan Xu, Xia Wang, Tao-Yang Wang, Zheng-Zhan Lin, Yong-Jian Hu, Zhong-Lin Huang, Xian-Jun Yang, Ping Xu

**Affiliations:** 1 Department of Pharmacy, Xinxiang Medical University, Xinxiang 453003, Henan, China; 2 College of Medical Laboratory, Xinxiang Medical University, Xinxiang 453003, Henan, China; 3 Henan Key Laboratory of Medical Tissue Regeneration, Xinxiang Medical University, Xinxiang 453003, Henan, China; 4 International Joint Research Laboratory for Recombinant Pharmaceutical Protein Expression System of Henan, Xinxiang Medical University, Xinxiang 453003, Henan, China

**Keywords:** flavonoids of *Rosa roxburghii* Tratt, radioprotective effect, mice thymus, ROS/DNA, PARP-1/AIF

## Abstract

This study aimed to evaluate the role of FRT in ROS/DNA regulation with or without PARP-1 in radiation-injured thymus cells. The administration of FRT to PARP-1-/- (KO) mice demonstrated that FRT significantly increased the viability of thymus cells and decreased their rate of apoptosis through PARP-1. Radiation increased the levels of ROS, γ-H2AX and 53BP1, and induced DNA double strand breaks. Compared with wild type (WT) mice, levels of ROS, γ-H2AX and 53BP1 in KO mice were much less elevated. The FRT treatment groups also showed little reduction in these indicators in KO mice compared with WT mice. The results of the KO mice study indicated that FRT reduced ROS activation through inhibition of PARP-1. Furthermore, FRT reduced the concentrations of γ-H2AX by decreasing ROS activation. However, we found that FRT did not regulate 53BP1, a marker of DNA damage, because of its elimination of ROS. Levels of apoptosis-inducing factor (AIF), exhibited no significant difference after irradiation in KO mice. To summarize, ROS suppression by PARP-1 knockout in KO mice highlights potential therapeutic target either by PARP-1 inhibition combined with radiation or by treatment with a drug therapy alone. AIF-induced apoptosis could not be activated in KO mice.

## INTRODUCTION

Poly (ADP-ribose) polymerase-1 (PARP -1) is the most abundant isoform in the PARP gene family and participates in the DNA base excision repair system [1]. PARP-1 is implicated in many cellular functions, including DNA repair, regulation of post-transcriptional gene expression and inflammation, and cell death [2–4]. PARP-1 is recruited to the site of DNA damage to facilitate DNA repair and ensure cell survival [5–7]. PARP-1 can be activated by damaged DNA and catalyzes the cleavage of NAD^+^ into nicotinamide and ADP-ribose to form long branches of ADP-ribose polymers on target proteins [8]. However, overactivation of PARP results in the concomitant depletion of its substrate NAD^+^ and consequently ATP. This failure in energy supply results in cell dysfunction and can culminate in necrosis [9, 10]. PARP-1 can be activated by high glucose or hyperglycemia in endothelial cells [11–13]. High glucose or hyperglycemia increases oxidative stress and induces DNA damage. PARP-1 overactivation contributes to the development of morbidity through two mechanisms [14]. Thus, expression of PARP-1 may be related to oxidation state. Oxidative stress promotes the formation of large numbers of DNA breaks which induces over-activation of PARP-1, resulting in apoptotic and necrotic cell death [15]. PARP inhibition reduces ROS-induced cell death, suppresses production of mitochondrial reactive oxygen species (ROS) and protects mitochondrial membrane potential in an ATF4 and MKP-1 dependent manner [16].

ROS originates from mitochondria and increases in concentration when exposed to ionizing radiation. As an agent of DNA injury, ROS causes damage to bases, single or double-strand breaks (SSB/DSB) and stimulates mitotic cell cycle arrest [8, 17, 18]. Recently, it has been shown that ROS-mediated DNA damage triggers activation of PARP-1 and subsequent cell death [19].

However, the specific mechanism of radioprotection in KO mice has been poorly reported. This study further explores the intrinsic relationship between PARP-1 and ROS/DNA in a KO mouse model of radiation damage.

PARP-1-mediated cell death has many characteristics that are associated with apoptotic cell death, for example, apoptosis-inducing factor (AIF) to be transferred from mitochondria to the nucleus [20, 21]. Sphingosine and radiation have been shown to synergistically enhance caspase-independent apoptotic cell death in radiation-resistant Jurkat T cell clones, establishing that PARP-1 activation is implicated in the nuclear translocation of AIF [22]. The pathway leading to cell death induced by PARP-1 activation is an important mechanism of AIF-mediated apoptosis and AIF appears to be the key mediator of PARP-1 in downstream cell death [23]. When the level of DNA damage is serious, PARP-becomes over-activated and consumes cellular energy (NAD, ATP) in the process of autologous poly-ADP nucleosylation, which causes AIF to move to the nucleus to mediate non-caspase-dependent apoptosis [24]. The transfer of AIF from mitochondria to the nucleus is a necessary condition for PARP-1-mediated cell death. We also found that PARP-1 activation is involved in the nuclear translocation of AIF and the subsequent cell death of radiation-resistant Jurkat clones. Pretreatment with PARP-1 inhibitor or transfection with PARP-1 siRNA has been demonstrated to effectively inhibit AIF translocation from mitochondria, nuclear aggregation and subsequent cell death induced by combined treatment [25].

The flavonoid fraction of the fruits of *Rosa roxburghii* Tratt (FRT), which has excellent antioxidant properties, has been extracted with a purity of 73.85%, its principal components consisting of catechin (34.26%) and quercetin (2.97%) [26]. Flavonoids have been demonstrated to exhibit significant radioprotective capabilities [27]. Previous studies have shown that FRT reduces apoptosis by regulating caspase 3/8-10, AIF and PARP-1, with inflammatory reactions suppressed through regulation of ICAM-1, IL-1α/IL-6β and TNF-α/NF-КB, thereby enhancing radiation protection [28]. The aim of this study was to explore the effect of FRT on KO mice including the relationship between PARP-1 and ROS, also ROS and DNA following their exposure to radiation.

## RESULTS

### FRT reduced radiation damage through PARP-1 *in vivo* and *in vitro*

**Figure 1:**
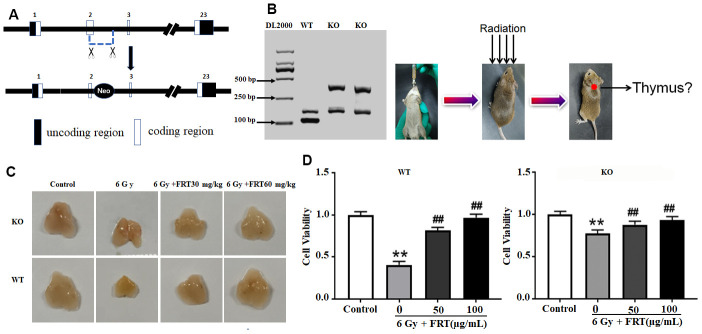
**FRT protected against radiation damage through PARP-1 both *in vivo* and *in vitro*.** (**A**) PCR was used to identify PARP-1 gene in KO mice; (**B**) Animal model of radiation injury induced by 6 Gy irradiation in mice; (**C**) Mice were administered FRT orally for 4 days prior to irradiation. The thymus was harvested 4 days after irradiation and the tissue appearance observed by using a digital camera (n=5). (**D**) Thymus cells were prepared from WT mice and KO mice and irradiated with a dose of 6 Gy. FRT of 50 and 100 μg/mL were provided to cells 2 h prior to irradiation. The CCK-8 method was used to measure cell viability 6 h after irradiation. Data was expressed as mean ±SD, n=5. (** P<0.01 compared with normal group; ## P< 0.01 compared with radiation group).

A promoterless neomycin gene replaced part of exon 2 and intron 2. The neomycin sequences were fused in-frame to the coding sequence of the PARP-1 gene. DNA was extracted from the tip of the mouse tails, and then subjected to PCR amplification and agarose electrophoresis. PARP-1 mice had a band at 350bp, WT mice had a band at 112bp, and 160bp was a non-specific amplified band (Figure 1A). A mouse model of radiation damage was successfully established in accordance with previous articles published by our team (Figure 1B). The thymus in the mouse is very sensitive to radiation injury. After 6 Gy irradiation, the volume of each thymus from the mice had severely atrophied, their size clearly decreased. Compared with WT mice, the volume of the thymus in KO mice decreased less than that those of normal mice. The difference between the FRT group and radiation groups was smaller than observed in WT mice. Therefore, FRT reduced the degree of atrophy of the thymus caused by radiation through the action of PARP-1, delivering good protection against radiation exposure to the mouse thymus (Figure 1C). The viability of thymus cells in the radiation group of WT mice was significantly lower than that of the normal group, but the viability of cells from KO mice was only slightly lower than that of normal group. The difference between the FRT group and radiation group was significantly reduced, compared with that of WT mice (Figure 1D). The results indicated that FRT increased the viability of thymus cells by regulating PARP-1.

### FRT improved morphological structure and reduced apoptosis of cells due to PARP-1 in thymus tissue

**Figure 2:**
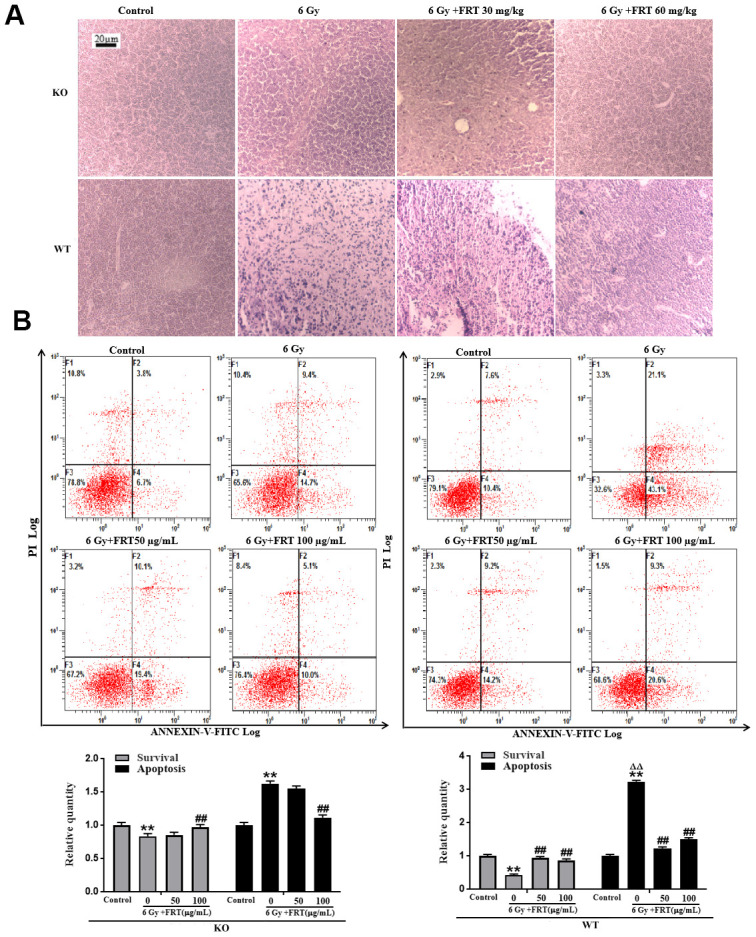
**FRT significantly improved the histopathological change of thymus and reduced the rate of apoptosis after radiation by PARP-1.** (**A**) WT and KO mice were randomly divided into 4 groups according to their body weight ① Normal group, ② Radiation group, ③ Radiation +FRT 30 mg/kg, ④ Radiation +FRT 60 mg/kg). Mice were given different doses of FRT by gastrointestinal administration for 4 days, and were irradiated at the dose of 6 Gy. Thymus was removed 4 days after irradiation. The tissues were fixed overnight with 4% paraformaldehyde, and then embedded in paraffin. After sectioning, the tissues were stained with hematoxylin and eosin (n=5). (**B**) Thymus cells were pretreated with or without FRT (50 and 100 μg/mL) prior to 6 Gy irradiation. Cells were harvested, and apoptosis was assessed by FCM (staining with both Annexin V-FITC and PI) 6 h after irradiation. The percentages of survival cells were compared (** *P*<0.01 compared with normal group; ## p < 0.01 compared with radiation group; ΔΔ *P*<0.01 compared with the KO mouse radiation group). PARP-1 knockout in mice can reduce apoptosis rate of thymus cells after radiation injury. Data was expressed as mean ±SD, n=5.

In mice where the PARP-1 gene was knocked out, damage to the thymus, as observed by histopathology, was significantly reduced after radiation, with no apparent damage to the thymus tissue in the radiation group. Compared with the radiation-only group, the reduction in damage to tissues from radiation as a result of administration of FRT was not as significant as that observed in WT mice (Figure 2A), indicating that FRT significantly reduced damage to the thymus through PARP-1, as demonstrated histopathologically. Similar findings were detected in the spleen and liver (Supplementary Figure 1A, 1B).

Apoptotic cells in the radiation group, measured by FCM, increased in number significantly, with radiation-induced apoptosis significantly reduced after administration of FRT. However, in the KO mice, apoptosis of the thymus cells following exposure to radiation decreased significantly compared with radiation group in WT mice. The difference in apoptosis in the thymus cells in the FRT group compared with the radiation group was relatively smaller. In summary, the results demonstrated that FRT reduced the apoptosis of thymocytes caused by radiation because of regulation by PARP-1 (Figure 2B and Supplementary Figure 2).

### FRT scavenged ROS after exposure to radiation due to PARP-1 *in vivo* and *in vitro*

**Figure 3:**
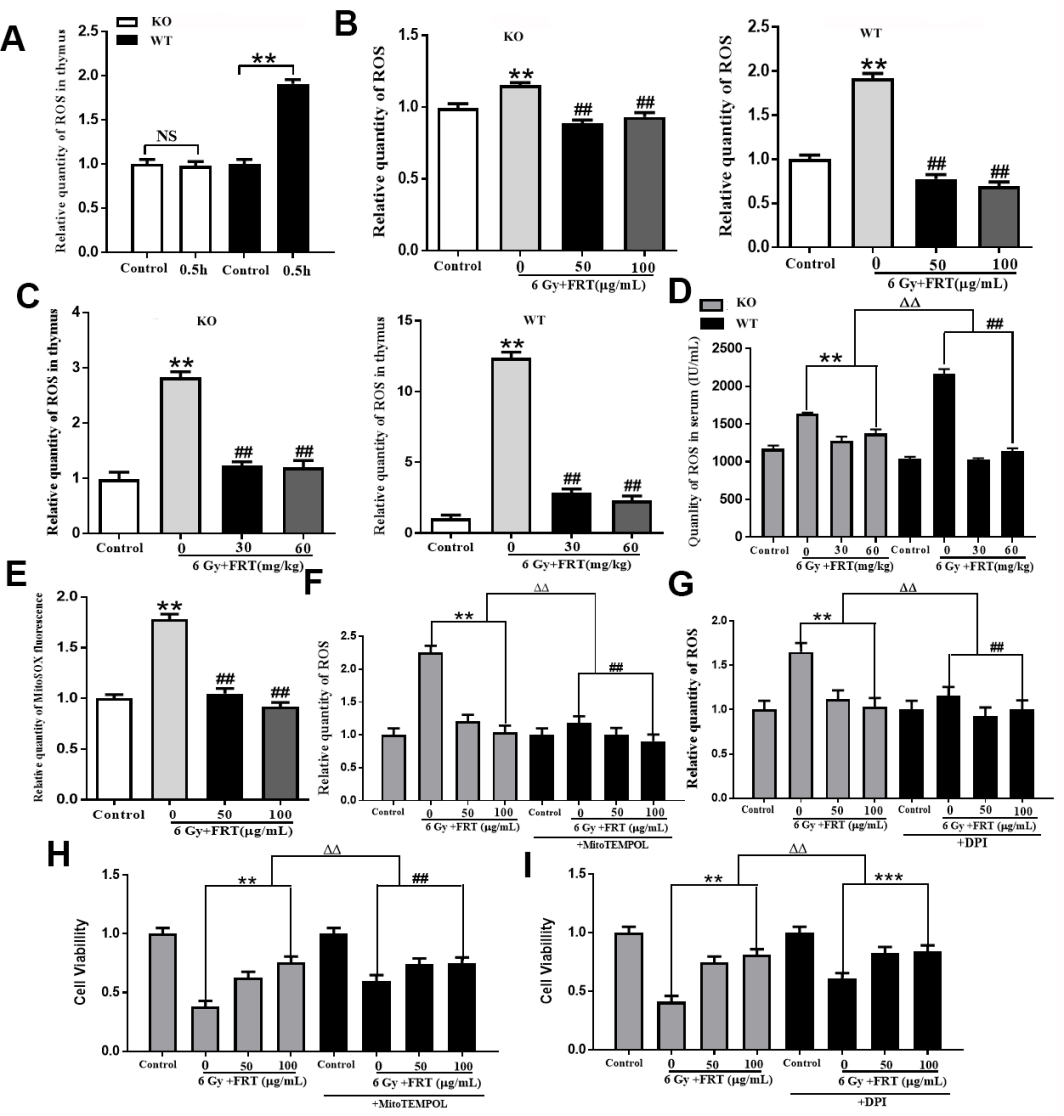
**Effect of FRT on scavenging intracellular ROS due to PARP-1**. (**A**) Representative flow cytometry histogram illustrating the radiation-induced change in ROS levels as detected by the DCFH-DA probe in thymus cells 0.5 h after radiation. (NS, not significant; ** *P* <0.01). Data was expressed as mean ±SD, n=5. (**B**) ROS levels in thymus cells measured by DCFH-DA probe. The thymus cells were irradiated at a dose of 6 Gy 2 hours after pretreatment with FRT (50 and 100 μg/mL). Cells were collected 0.5 h after irradiation. (** *P*<0.01 compared with normal group; ## *P*<0.01 compared with radiation group). Data was expressed as mean ±SD, n=5. (**C**) ROS levels in mice measured using DCFH-DA probe. Mice were pretreated with FRT (30 and 60 mg/kg) 4 days prior to 6 Gy irradiation, then sacrificed and thymus harvested 4 days after radiation. (**P <0.01 compared with normal group; ## *P*<0.01 compared with radiation group). Data was expressed as mean ±SD, n=5. (**D**) ROS levels in serum were quantified by ELISA 4 days after irradiation. (** *P*<0.01; ## *P*<0.01; ΔΔ *P*<0.01). Data was expressed as mean ±SD, n=5. (**E**) Representative luciferase labeling instrument displaying MitoSOX fluorescence in thymus cells under different conditions. Mitochondrial ROS as measured with the MitoSOX probe (**P<0.01 compared with normal group; ## *P*<0.01 compared with radiation group). Data was expressed as mean ±SD, n=5. (**F**) Thymus cells were treated with FRT 2 h before irradiation. The cells were incubated with MitoTEMPOL (50 μM) inhibitor for 1 h before irradiation then collected by centrifugation 0.5 h after irradiation. Flow cytometry was used to measure intracellular ROS levels. (** *P*<0.01; ## *P*<0.01; ΔΔ *P*<0.01). Data was expressed as mean ±SD, n=5. (**G**) Thymus cells were pretreated with FRT for 2 h before irradiation. The cells were incubated with DPI (10 μM) for 1 h prior to irradiation and 0.5 h after irradiation. The effect of DPI on ROS levels in thymus cells after irradiation was measured by flow cytometry. (** *P*<0.01; ## *P*<0.01; ΔΔ *P*<0.01). Data was expressed as mean ±SD, n=5. (**H**) The survival fraction of thymus cells treated with or without ROS inhibitor in mitochondria (MitoTEMPOL, 50 μM). Cell viability was measured 6 h after radiation by CCK-8 assay. MitoTEMPOL reduced the sensitivity of thymus cells to radiation and enhanced the viability of thymus cells (** *P*<0.01; ## *P*<0.01; ΔΔ *P*<0.01). Data was expressed as mean ±SD, n=5. (**I**) Proportion of surviving thymus cells treated with or without inhibitor of NAPDH oxidase (DPI, 10 μM). Cell viability was measured 6 h after radiation by CCK-8 assay. DPI reduced the sensitivity of thymus cells to radiation and enhanced their viability (** P<0.01; ## P<0.01; ΔΔ P<0.01). Data was expressed as mean ±SD, n=5.

After the mouse thymus cells were exposed to 6 Gy irradiation, their levels of ROS in two breeds of mice were measured using a reactive oxygen species detection kit after 30 min. The results demonstrated that ROS levels increased significantly half an hour after irradiation of WT mice, while levels of ROS in KO mice did not change much. This result suggested that inhibition of PARP-1 might protect mice from radiation by decreased ROS levels caused by radiation (Figure 3A and Supplementary Figure 3A).

The levels of ROS in the thymus cells increased significantly due to irradiation. Treatment with 100 μg/mL and 50 μg/mL FRT significantly reduced levels of ROS in the cells from WT mice. As to cells from KO mice, ROS levels did not change significantly after irradiation. The change in ROS levels due to FRT was not as significant as in the WT mice (Figure 3B and Supplementary Figure 3B). ROS levels in the thymus increased significantly due to irradiation, and the radiation group was 12.31 times that of the normal group in WT mice, compared with 2.83 times in KO mice. Correspondingly, the reduction of ROS by 30 mg/kg and 60 mg/kg FRT in KO mice was far less significant than that of WT mice. These results demonstrate that PARP-1 plays an important role in maintenance of the levels of ROS in the thymus of irradiated mice (Figure 3C and Supplementary Figure 3C).

In the present study, ELISA was used to measure the concentration of ROS in the serum of mice. In the WT mice treated with 30 mg/kg and 60 mg/kg FRT, ROS concentration reduced significantly. However, in the KO mice, the levels in serum did not increase greatly after irradiation. The reduction in ROS due to FRT was less than that observed in the WT mice, indicating that knockout of the PARP-1 gene reduced the active oxygen species in serum, the reduction in ROS by FRT being related to PARP-1 (Figure 3D). Administration of FRT reduced the levels of ROS labeled by MitoSOX in mitochondria, demonstrating that the ROS in thymic cells reduced by FRT after irradiation was partially derived from mitochondria (Figure 3E).

To illustrate the effect of FRT on mitochondrial reactive oxygen, MitoTEMPOL, an inhibitor of mitochondrial reactive oxygen, was added to the thymus cell culture. The results indicated that intracellular ROS levels in thymus cells in the irradiated group were higher than those in the control group and in the irradiated FRT group the level was less than that in the irradiated group. After intervention with MitoTEMPOL, the reduction in ROS due to FRT was significantly reduced (Figure 3F and Supplementary Figure 3D).

To illustrate the effect of FRT on reactive oxygen induced by NADPH oxidase, DPI, an inhibitor of NADPH oxidase, was added to the thymus cell culture. The results demonstrated that the level of ROS in the thymus cells in the irradiated group was higher than that in the control group. Intracellular ROS levels in the irradiated FRT group were smaller than those in the irradiated group. After intervention with DPI, the reduction in ROS due to FRT was significantly reduced (Figure 3G and Supplementary Figure 3E).

In order to further verify the effect of FRT on ROS from mitochondria and NADPH oxidase, cell viability was measured after MitoTEMPOL and DPI were added. The cell viability of cultures with added MitoTEMPOL (Figure 3H) and DPI (Figure 3I) in the irradiation group was significantly higher than that in the irradiation thymus cells from radiation injury. It also demonstrated that FRT increased cell activity by removing ROS from mitochondrial and NADPH oxidase.

### FRT protected from radiation-induced DNA damage due to ROS in thymus tissue

**Figure 4:**
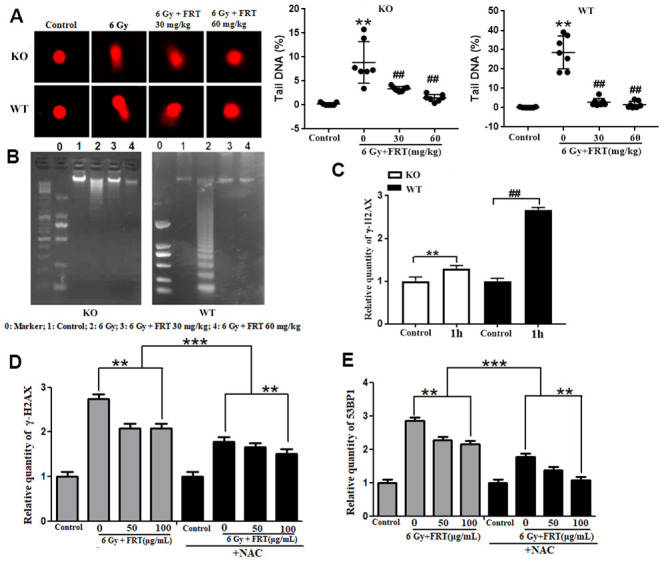
**Protective effect of FRT on radiation-induced DNA damage due to ROS in thymus tissue.** (**A**) Effect of FRT on radiation-induced DNA damage measured by comet assay in thymus tissue of KO mice (**P <0.01 compared with normal group; ## *P*<0.01 compared with radiation group). Data was expressed as mean ±SD, n=5. (**B**) Protective effects of FRT on radiation-induced DNA fragments measured by DNA Ladder assay in thymus tissue of KO mice. PARP-1 knockout reduced radiation-induced DNA fragmentation in irradiated thymus cells (n=5). (**C**) The thymus was removed from WT mice and KO mice then ground into a cell suspension prior to allocation into a control group or 6 Gy radiation group. Thymus cells were harvested 1h after irradiation and γ-H2AX levels were measured by flow cytometry (** *P*<0.01; ## *P*<0.01). Data was expressed as mean ±SD, n=5. (**D**) Thymus cells were treated with FRT and inhibitor of ROS (NAC, 0.5 mM) 2 h before irradiation. Intracellular 53BP1 levels were measured by flow cytometry 1 h after irradiation. (** P<0.01; *** P<0.01). Data was expressed as mean ±SD, n=5. (**E**) Thymus cells were treated with FRT and inhibitor of ROS (NAC, 0.5 mM) 2 h before irradiation. Intracellular γ-H2AX levels were measured by flow cytometry 1 h after irradiation. (** P<0.01; *** P<0.01). Data was expressed as mean ±SD, n=5.

Following identification of the relationship between PARP-1 and ROS, the relationship between ROS and DNA required clarification. In comparison with KO mice, the tail in the comet assay of WT mice in the irradiated group was longer, indicating greater DNA damage. The tail in the FRT intervention group was shorter than in the radiation group (Figure 4A, Supplementary Figure 4A, 4B). In WT mice, radiation-induced DNA double bond breaks and fragmentation increased more than in KO mice, exhibiting a more pronounced trapezoidal band (Figure 4B). The phosphorylation level of γ-H2AX in thymus cells within an hour of irradiation was measured by flow cytometry. Compared with KO mice, levels of γ-H2Ax in the thymus cells of WT mice were significantly higher, indicating that knockout of PARP-1 protected DNA (Figure 4C and Supplementary Figure 4C, 4A).

The mechanism of action of FRT in relation to the protection of DNA is currently unclear, although given the relationship between PARP-1 and ROS, we should consider that it involves interaction of ROS with DNA. The levels of γ-H2AX in thymus cells in the irradiated group were significantly higher than in the control group. The intracellular γ-H2AX level in the irradiated NAC group was lower than that in the irradiated group (without NAC). In the NAC groups, the effect of FRT on the reduction of γ-H2AX was less significant than in groups without NAC. The results above indicated that FRT reduced γ-H2AX by eliminating ROS (Figure 4D and Supplementary Figures 4, 3B). 53BP1 is another important regulatory factor in response to double-strand breaks. Compared with the control group, intracellular 53BP1 levels in thymus cells in the irradiation group were significantly higher than those in the normal group, whereas the level in the irradiated NAC group was lower than in the irradiated group (without NAC). In the NAC groups, the effect of FRT on the reduction of 53BP1 was as significant as in the groups without NAC (Figure 4E and Supplementary Figures 4, 3C). The results above indicated that the action of FRT was principally towards a reduction in 53BP1, not through the elimination of ROS.

### FRT played a role in radiation protection by regulating PARP-1/AIF

**Figure 5:**
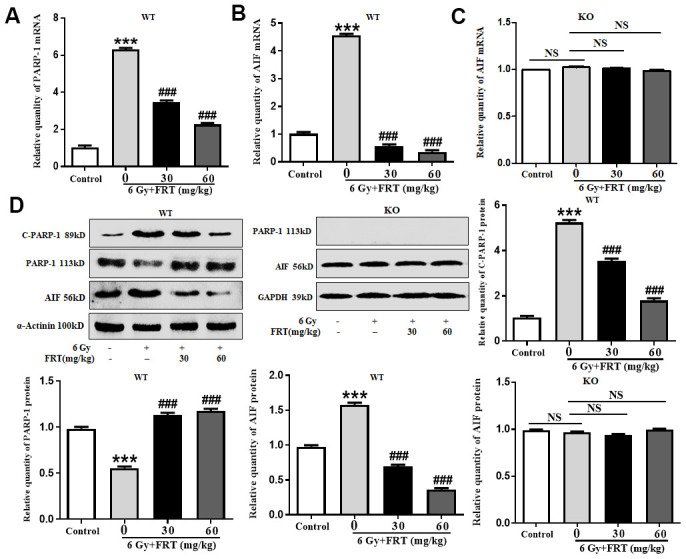
**FRT played a role in radiation protection by regulating PARP-1/AIF.** KO mice and WT mice were pretreated with FRT, then after 4 days irradiated with a dose of 6 Gy. Thymus tissue was removed 4 days after radiation. (**A**) RNA was extracted using a homogenizer and the expression of PARP-1 gene was quantified using fluorescent quantitative PCR. PARP-1 gene expression was increased after irradiation of WT mice. FRT was effective in inhibiting activation of PARP-1 after irradiation (*** *P*<0.01 compared with the control group; ### *P*<0.01 compared with radiation group). Data was expressed as mean ±SD, n=5. (**B**) AIF gene expression increased after irradiation in WT mice. FRT was effective in inhibiting activation of AIF after irradiation (*** *P*<0.01 compared with the control group; ### *P*<0.01 compared with radiation group). Data was expressed as mean ±SD, n=5. (**C**) In the thymus tissues of KO mice, there was no significant difference in AIF gene expressions between the control, radiation group and FRT drug groups (NS, not significant). Data was expressed as mean ±SD, n=5. (**D**) Proteins in thymus tissues were extracted by RIPA lysis buffer. Protein expressions of PARP-1 and AIF, as measured by Western blot analysis, increased after irradiation in WT mice. FRT effectively inhibited the activation of PARP-1 and AIF after irradiation. In the thymus tissues of KO mice, there was no significant difference in AIF protein expressions between the control, radiation group and FRT drug groups (NS, not significant; *** *P*<0.01 compared with the control group; ### *P*<0.01 compared with radiation group). Data was expressed as mean ±SD, n=5.

In the WT mice, compared with the normal group, the expressions of PARP-1 and AIF mRNA in the radiation group increased significantly. Compared with the radiation group, 30 mg/kg and 60 mg/kg FRT significantly down-regulated the expressions of related genes (Figure 5A, 5B). In KO mice, there was no significant difference in the mRNA expression of AIF in each experimental group (Figure 5C).

Following the exposure of WT mice to radiation, the protein expressions of C-PARP-1 and AIF increased, but decreased significantly when treated with 30 mg/kg and 60 mg/kg FRT. These data suggested that FRT protected cells from radiation damage through the PARP-1/AIF pathway. KO mice did not express PARP-1 protein, and there was no significant difference in the protein expressions of AIF in any group, So it can be concluded that PARP-1 knockout inhibited AIF release, suggesting that PARP-1 was the upstream regulatory molecule of AIF (Figure 5D).

### FRT played a role in radiation protection by inhibiting PARP-1 activation and reducing AIF release

**Figure 6:**
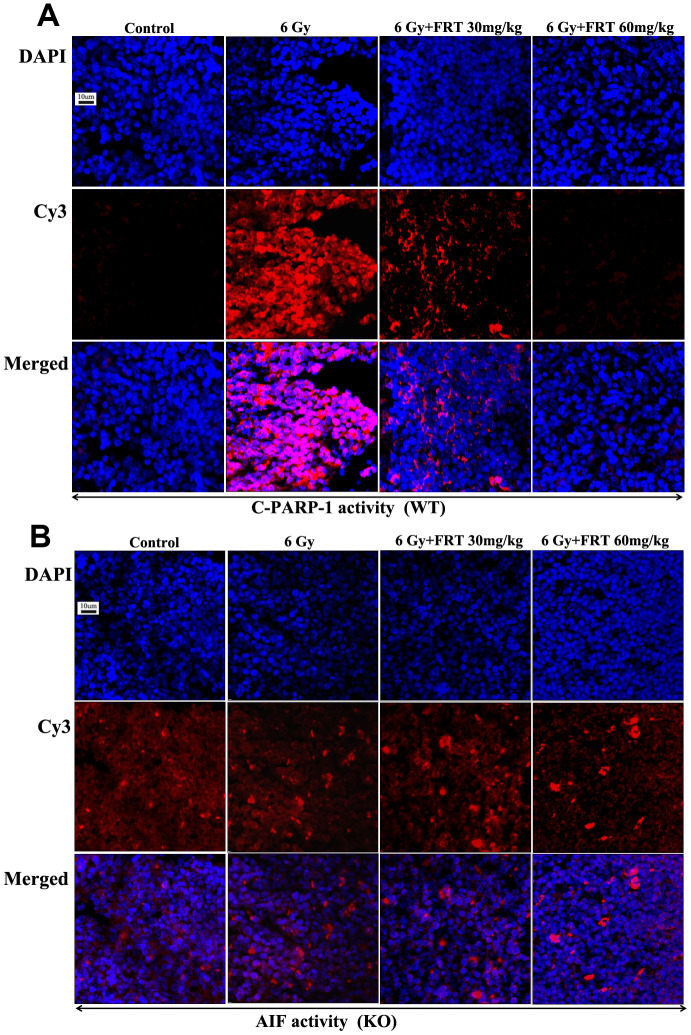
**FRT played a role in radiation protection by inhibiting PARP-1 activation and reducing AIF release.** (**A**, **B**) Expressions of C-PARP-1 and AIF in mouse thymus tissues measured by immunofluorescence. Paraffin-embedded specimens were sliced into sections (4 μm), stained with Cy3 labeled fluorescent secondary antibody (1:500) combined with primary antibody against specific proteins and with DAPI for nuclear staining, sealed with anti-fluorescence quencher, then observed immediately using confocal laser scanning microscopy (n=5).

The fluorescence intensity of C-PARP-1 in thymus tissues of WT mice following irradiation was significantly higher than that observed in the normal group. The expressions of C-PARP-1 decreased significantly after 30 and 60 mg/kg FRT treatment compared with that in the radiation group alone. So, treatment with 30 mg/kg FRT, 60 mg/kg FRT caused a slightly stronger inhibitory effect on C-PARP-1 after radiotherapy. Therefore, FRT inhibited the activation of PARP-1 and reduced the apoptosis of thymocytes (Figure 6A).

In KO mice, there was no significant difference in the expression of AIF fluorescence intensity in thymus tissues between the FRT drug groups and the radiation group. Therefore, FRT acted to protect against radiation by inhibition of PARP-1 cleavage to reduce the release of AIF (Figure 6B).

## DISCUSSION

The major finding of this study was that FRT could protect against radiation-induced DNA damage by regulating PARP-1/ROS/DNA both *in vivo* and *in vitro*. Our previous research results demonstrated that FRT inhibited pathway of apoptosis by downregulation of C-caspase-3 and C-PARP-1. In addition, we found that FRT attenuated intracellular ROS levels and scavenged OH•, DPPH and •O^2-^ *in vitro* [27]. However, our previous studies did not indicate whether FRT regulated ROS through PARP-1 to protect DNA. For the first time, this study demonstrated that FRT protected against radiation damage by regulating the PARP-1/ROS/DNA pathway.

PARP-1 inhibitor provides radio-sensitivity to tumor cells. ROS suppression by PARP-1 in muscle-invasive bladder cancer is a potential therapeutic target either for PARP-1 inhibitor combined with radiation or treatment with a drug alone [29]. In a KO mouse model, ROS levels increased with age and a significant number of genes linked to oxidative stress and ROS production were dysregulated compared with wild-type mice [30]. Interestingly, this study demonstrated that in a KO mouse model, ROS levels produced in the thymus after irradiation were lower than those produced in wild type mice. A major finding of this study is that PARP-1 knockout decreases oxidative stress by decreasing NAD(P)H oxidase-derived and mitochondrial-derived ROS in thymus cells after irradiation. Scavenging of ROS using MitoTEMPOL or inhibition of NAD(P)H oxidase with DPI significantly reversed the levels of ROS in irradiated mouse thymus cells. FRT (50 and 100 μg/mL) was administered 2 hours prior to irradiation with MitoTEMPOL (50 μM) or DPI (10 μM) inhibitor administered 1 hour prior to irradiation, which inhibited the release of ROS in thymus cells. After the use of a ROS inhibitor, the ability of FRT to reduce ROS was significantly reduced. It was suggested that FRT regulated ROS in these two pathways through PARP-1. Thus, PARP-1 causes different effects on the regulation of ROS in both normal and tumor cells after irradiation. Inhibition of PARP-1 significantly promotes the production of ROS in cancer cells after irradiation, which may be related to differences in cell type [31, 32].

DNA-damage is the principal process by which apoptosis is induced by irradiation of the cells. ROS acts as an agent to damage DNA by producing a series of DNA lesions, including base damage, single or double-strand breaks, DNA-DNA or DNA-protein crosslinks. Double-strand breaks are the most harmful form of DNA damage often resulting in cell death [33–35]. We have also proposed a novel mechanism explaining how FRT regulated ROS-DNA to protect against radiation damage. Excessive ROS can lead to DNA double strand breakage and DNA damage, ultimately resulting in oxidative damage [36]. H2AX is a subgroup of histone H2A, which is first phosphorylated under ionizing radiation, and the activated γ-H2AX reaches the highest level within 0.5 h after radiation. It plays an important role in the repair of DNA double strand breaks induced by radiation [37]. It was found that γ-H2AX could be used as a protein marker for the degree of DNA damage. Flow cytometry showed that the phosphorylation of H2AX around chromatin DNA double strand breaks was almost correlated with the number of DNA double strand breaks at 1:1 [38]. P53 binding protein 1 (p53-binding protein 1, 53BP1) plays an important role in the signal transduction of DSBs response to double strand break damage [39, 40]. In the early stage of DNA double strand fracture, H2AX rapidly is phosphorylated into gamma H2AX and aggregates to the DSB site, and then provides a platform to collect other DNA repair proteins (such as 53BP1, BRCA1, etc.) to the damage site [41]. Studies have found that gamma H2AX and 53BP1 have the function of joint localization under conditions such as ionizing radiation [42]. This study verified that levels of γ-H2AX and 53BP1, receptors of DNA damage, reflected this impairment. We also found that FRT reduced H2AX and 53BP1 by removing ROS, but the ability of FRT to reduce H2AX was significantly reduced after the addition of NAC. Surprisingly, after NAC was added, FRT significantly reduced expression of 53BP1, indicating that FRT protects DNA from radiation damage by γ-H2AX, not by 53BP1 through the removal of ROS.

**Figure 7:**
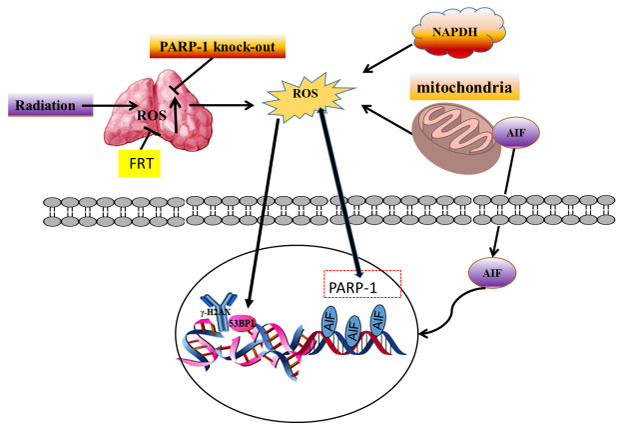
**FRT protected against radiation damage by regulating the PARP-1/ROS/DNA pathway, and by inhibiting the activation of PARP-1/AIF.**

In summary, the experimental model in this study provided mechanistic insight indicating interplay between PARP-1 function, ROS production and DNA damage. The radioprotection by FRT relied on PARP-1 to control increased ROS production to protect DNA. This study was the first to report that PARP-1 deletion can inhibit production of ROS after exposure to radiation and that FRT administration can protect DNA from radiation damage through PARP-1 regulation. Our results suggest that PARP-1 may be the principal target of FRT in protecting cells from radiation damage. PARP-1 and ROS are the two main participants in radiation injury, consistent with the role of PARP-1 inhibitors [25]. We have demonstrated that ionizing radiation combined with FRT can enhance radiation resistance through PARP-1-dependent cell death pathways by inhibiting ROS production and PARP-1 activation. Combined FRT and PARP-1 knockout treatment significantly reduced intracellular ROS levels and inhibited mitochondrial translocation of AIF (Figure 7). Taken together, PARP-1 inhibitor treatment for radiation damage may thus represent a promising biomarker-directed therapeutic or preventive strategy.

## MATERIALS AND METHODS

### Materials

Ko Mice 6 to 8 weeks old were purchased from the Jackson Lab (stock No. 002779) in the United States. WT mice purchased from Beijing Wei-tong-li-hua Experimental Animal Technology Co., Ltd. All mice were individually housed under specific pathogen-free facilities with standard environment, diet and water. All of the animal experiments were conducted in accordance with the Guide for the Care and Use of Laboratory Animals, and the study was approved by the Animal Center of Xinxiang Medical University.

Thymus cells were cultured in suspension in RPMI-1640 medium containing 10% fetal bovine serum, 100 U/mL penicillin and 100 U/mL streptomycin at 37 °C in a humidified atmosphere containing 5% CO_2_.

PARP-1 antibody was purchased from Wan-Lei Biotechnology Co., Ltd (WL01932, Shenyang, China). Cleaved-PARP-1 antibody was from Elabscience Biotechnology Co., Ltd (E-AB-30080). AIF was obtained from CST Corp. (4642, MA, USA). Secondary antibodies included goat anti-rabbit IgG-Cy3 (A0516, Bi-Yun-Tian Biotechnology Co., Ltd, Shanghai, China) and horseradish peroxidase (HRP)-labeled antibody (CST, 7074S, MA, USA). A rapid identification kit for mouse tail genotyping was purchased from Bi-Yun-Tian Biotechnology Co., Ltd. (D7283S, Shanghai, China). ELISA kits for the analysis of serum ROS were provided by Wuhan Hualianke biotechnology Co., Ltd (DER0297, Wuhan, China). Flow cytometry (FCM) Annexin V-FITC apoptosis detection kits were purchased from Bi-Yun-Tian Biotechnology Co., Ltd (C1062M, Shanghai, China). 53BP1 (ab36823) and H2AX (ab11174) antibodies were purchased from Abcam, UK, and stored at -20°C. An Alexa-488 secondary antibody (ab150077) was purchased from Abcam, UK, and stored in the dark at -20°C.

Diphenyleneiodonium chloride (DPI, Selleck Chemicals, S8639) was dissolved in DMSO at a concentration of 10 mM and stored at -20 °C as a stock solution then diluted to 10 μM when required for use. N-acetylcysteine (NAC, Biyuntian Biotechnology, S0077) and MitoTEMPOL (Abcam, ab144644) were dissolved in ddH_2_O and stored at -20 °C then used experimentally at 0.5 mM and 50 μM, respectively. The compounds were stored in the dark separately, to avoid excessive numbers of freeze thaw cycles. Prior to each use, the ROS probe dichlorodihydro-fluorescein diacetate (DCFH-DA, Biyuntian Biotechnology, S0033) was diluted 1:1000 in serum-free medium to a final concentration of 10 μM. MitoSOX (Thermo Fisher Scientific, M36008) was dissolved in DMSO to a concentration of 5 mM then diluted to 5 μM with HBSS. The following reagents were used to quantify gene expressions, RNAiso Plus reagent (Takara Bio Inc.), ReverTra Ace qPCR RT Master Mix kit (Toyobo Co., Ltd. Life Science Department, Osaka, Japan) and SYBR1 Green Real-time PCR Master Mix kit (Toyobo Co., Ltd.).

### Irradiation

Cells were irradiated *in vitro* at room temperature at a dose rate of 100 cGy/min for a total dose of 6 Gy [27]. Cells in the treatment group were supplemented at concentrations of 50 μg/mL and 100 μg/mL for 2 hours prior to irradiation.

For the *in vivo* studies, animals were restrained in bespoke boxes [43] and then exposed to 6 Gy of total-body radiation at a dose rate of 100 cGy/min. FRT was administered orally for 4 days at a dose of 30 mg/kg or 60 mg/kg prior to irradiation. On the 4^th^ day following irradiation, the mice were sacrificed and each thymus harvested.

### Thymus appearance and shape

After 4 days of irradiation, the mice were sacrificed and the appearance and shape of each thymus recorded using a digital camera.

### Histological analysis of thymus tissue (in addition to liver and spleen)

Pre-adapted mice were divided into normal (non-irradiated), irradiated (untreated), and drug treatment groups. All drugs were administered orally. The normal and irradiation groups were administered distilled water. The treatment group was administered FRT at doses of 30 or 60 mg/kg prior to irradiation for 4 days. Mice were sacrificed 4 days after irradiation (6 Gy). Excised tissue was fixed in 4% paraformaldehyde, dehydrated in an ascending gradient of ethanol, embedded in paraffin, sliced into 4 μm-thick sections, stained using hematoxylin and eosin (HE) then examined using a light microscope (Nikon, Tokyo, Japan).

### Detection of cell activity and mitochondrial ROS using plate reader

### Cell viability assay

Two hundred μL of thymus cells (1×10^6^ cells/mL) were inoculated into each of the wells of a 96-well plate and grouped according to the experimental design, with repeats. After incubation with FRT for 2 hours, MitoTEMPOL(50 μM) or DPI(10 μM) were added to cells 1 h before radiation. The cells were irradiated using a single dose of 6 Gy. Six hours after irradiation, 10 μL CCK-8 was added to the cells and incubated at 37°C for 4 hours. The absorbance at 450 nm was measured using a plate reader.

### Mitochondrial ROS quantity

The cells were collected and incubated with 600 μL MitoSOX 0.5 h after radiation and washed with HBSS (37 °C in advance) at 37 °C for 10 min. The cells were washed with HBSS (37 °C in advance) for three times. Finally, the fluorescence intensity of 200 μL cell suspen-sion per hole was detected by a plate reader (excitation/ emission maxima of approximately 510/580 nm).

### Flow cytometry assays (FCM)

Annexin V-FITC was used to detect apoptotic cells. Thymus cells (1×10^6^ cells/mL) were inoculated into 6-well plates in RPMI-1640 medium and divided equally into normal, radiation, FRT 50 μg/mL and FRT 100 μg/mL groups. Cells were incubated with treatment drugs for 2 hours then irradiated with 6 Gy radiation. After six hours, the cells in each group were centrifuged at 1000g for 5 minutes. After discarding the supernatant, the cells were gently resuspended in PBS then counted. The cells were pelleted and washed three times with cold PBS then gently resuspended in 195 μL Annexin V-FITC binding solution. Ten μL Annexin V-FITC and 5 μL propidium iodide (PI) dye were then carefully added prior to incubation at room temperature for 15 minutes in the dark. Apoptosis was detected by flow cytometry (FCM).

To evaluate the formation of intercellular ROS, mouse thymus cells were collected 0.5 h after radiation (50 μM MitoTEMPOL or 10 μM DPI were added to cells 1 h before radiation), then loaded with the fluorogenic probe. DCFH-DA, which was diluted 1:1000 with serum-free medium to achieve a final concentration of 10μM. The cells were suspended in diluted DCFH-DA then incubated at 37°C for 20 minutes. The cells were inverted every 3-5 minutes to ensure they had full contact with the probe. The cells were washed three times with serum-free cell culture medium. Rosup (final concentration 50 μg/mL) was added to the positive control well only. Fluorescence from the fluorogenic probe was detected by FCM.

DNA damage induced by radiation was assessed by FCM as follows. Cells were collected 1 h after radiation (0.5 mM NAC or FRT were added 2 h before radiation), washed twice with PBS then fixed in 80% methanol for 10 minutes. The methanol was removed by centrifugation of the cells which were washed once with PBS then permeabilized in 0.3% Triton X-100 for 20 minutes. The Triton X-100 was removed by centrifugation and the cells were blocked using 10% sheep serum for 1 hour. The sheep serum was removed by centrifugation and 200 μL H2AX/53BP1 antibody (1:2000/1:1000) added to the cells which were incubated at 37°C for 2 hours. The cells were then washed twice with PBS after which they were incubated at room temperature for 1 hour with 200 μL Alexa-488 antibody (diluted 1:3000). One mL PBS was added to each sample then 20 μL of 1 mg/mL RNase and 10 μL of 500 μg/mL PI added. After 10 minutes of incubation, the cells were analyzed by FCM (BD Biosciences).

### Detection of ROS in serum using ELISA

About 1 mL blood collected from the eyeballs of mice was clotted, from which serum was collected. Ten wells of the ELISA plate were used to create a standard curve. After dilution, the quantity of standard in each well was 50 μg, with final concentrations of 480 μg/mL, 320 μg/mL, 160 μg/mL, 80 μg/mL and 40 μg/mL.

Forty μL of sample diluent were added to all the wells of the enzyme-coated plate to be tested and then 10 μL sample were added (except for blanks) and the plate was gently shaken then mixed. Fifty μL of enzyme-labeled reagent were added to each well, except for blanks. The plate was heated for 30 minutes at 37°C after sealing, after which the plate sealing film was carefully removed, the liquid discarded and the plate dried. Each well was filled with detergent which was discarded after 30 seconds, a procedure that was performed a total of five times. fifty μL chromogenic agent A and chromogenic agent B were added in turn to each well and the plate was then shaken gently and placed in the dark at 37°C for 10 minutes. Finally, 50 μL of stop reagent were added to each well and then the absorbance of each was measured at 450nm.

### Identification of PARP-1 gene in mouse tail tissue

Scissors and tweezers were washed in 70% ethanol prior to experimentation. The final 0.2~1 cm of the tails of experimental mice were placed in 100 μL digestive fluid (preparation: 960 μL Extraction Solution + 40 μL enzyme mix), making sure the tip was completely immersed in solution. The samples were placed in the PCR instrument at 55 °C and incubated for 15 min, then at 95 °C for 5 min. One hundred μL of stop solution were added to each sample which were then vortex mixed. The undigested tissue was removed by centrifugation and then agarose gel electrophoresis performed after PCR amplification (94 °C, 3min; 94 °C, 30S, 35 cycles; 55 °C, 30S; 72 °C, 8.46S; 72 °C, 10 min; 4 °C, hold).

### Comet assay (alkaline single cell gel electrophoresis, ASCGE)

Thymus cells (1×106cells/mL) from mice that had been treated with the experimental drug and irradiated were suspended in 0.4% low melting point agarose and transferred to frosted glass slides pre-coated with 0.8% normal melting point agarose, followed by an additional layer of 0.4% low melting point agarose to cover the surface. For each layer of agarose, the slides were placed in a refrigerator at 4 °C for 20 min to solidify the agarose gel. The slides were then immersed in freeze-cracking buffer (2,500 mM NaCl, 100 mM Na2-EDTA, 100 mM Tris base at pH 10, 1% sarcosine Na, l% Triton X-100 in 10% DMSO) then soaked at 4 °C for 1.5 h. The slides were then transferred to a horizontal electrophoresis tank and the DNA unfolded in frozen electrophoresis buffer (30 mM NaOH, 1 mM Na2-EDTA) for 20 min. Electrophoresis was performed at 19V for 20 min at 4 °C. After electrophoresis, the slides were placed in neutralizing buffer (400 mM Tris-HCI, pH 7.5) at 4 °C for 15 min then stained with propionate iodide. Images of the comet were obtained using a laser scanning confocal microscope.

### DNA fragmentation assay (DNA ladder)

Cells (1×106 cells/mL) were centrifuged (1800 rpm, 4°C, 5 min) then washed twice with PBS. Fifty μL lysate were added to the pelleted cells which were then gently resuspended and centrifuged, the supernatant being retained. Five μL enzyme A were added and the mixture incubated for 15 minutes at 37°C after which 5 μL enzyme B were added, the tube gently mixed and incubated at 50°C for 30 min. Forty μL of ammonium acetate and 200 μL of cold ethanol were then added, the mixture stirred and reacted at 20°C for 30 minutes. Each tube was then centrifuged (12000 rpm, 4°C, 10 min) until DNA precipitation was achieved. Ten μL Tris-EDTA (TE) buffer were added after each DNA precipitate had been dried at room temperature for 10 min. Ten μL DNA sample were mixed with 2 μL buffer which were then analyzed by electrophoresis in a 1.5% agarose gel at a voltage of 4V/cm.

### Western blot analysis

Thymus tissue was removed and cut into fine fragments. Lysis buffer was added to the tissue at a ratio of 150-250 μL lysis buffer per 20 mg tissue and then homogenized until fully dispersed. The suspension was centrifuged at 10000-14000 g for 5 minutes, from which the supernatant was retained and the precipitate discarded. The protein concentration was quantified using a BCA assay and protein levels of AIF and PARP-1 (C-PARP-1) determined by Western blotting. The density of protein bands was ascertained using image J software.

### Quantitative real-time polymerase chain reaction (RT-PCR)

Total RNA was extracted from 50 mg thymus tissue by the addition of 1 mL RNAiso Plus reagent. The corresponding cDNA was created from 1 μg of the RNA from each sample using a ReverTra Ace qPCR RT Master Mix kit, in accordance with the manufacturer’s instructions, incubating at 37°C for 15 min, 50°C for 5 min and 98°C for 5 min. Quantitative PCR was conducted using an ABI5500 RT-PCR system with a SYBR1 Green Real-time PCR Master Mix kit, using the following procedure: 95°C for 1 min, 40 cycles of 95°C for 15 s, 60°C for 30 s and 72°C for 45 s. The following primer sequences were used. PARP-1-F: AGGACGCTGTTGAGCACTTC and PARP-1-R: GCTTCTTTACTGCCTCTTCG; AIF-F: CAGCCAGACAGCATCATCCA and AIF-R: TAAAGCCGCCCATAGAAACC; GAPDH-F: AGGTCGGTGTGAACGGATTTG and GAPDH-R: TGTAGACCATGTAGTTGAGGTCA. mRNA levels of the target genes were calculated relative to the endogenous control GAPDH, using the 2^-ΔΔCT^ method.

### Detection of C-PARP-1 and AIF by immunofluorescence

Each thymus was harvested 4 days after irradiation, fixed in 4% paraformaldehyde at 4°C for overnight then embedded in paraffin. The tissue blocks were sliced to 4 μm with a paraffin slicer then individual sections placed on polylysine-treated slides, dewaxed by placing in xylene and a gradient of alcohol concentrations. The sections were then placed in a 0.01M sodium citrate solution at pH=6.0 for antigen retrieval. The thymus sections were permeabilized by treatment with 0.3% Triton X-100 for 20 minutes, washed 3 times with 0.01 M PBS for 5 minutes, then blocked using 10% sheep serum in a wet box for 30 min at 37°C. The appropriate volume of C-PARP-1 and AIF primary antibodies were pipetted onto the tissue sections which were placed in a humid chamber at 4°C overnight. The primary antibody was then washed off with PBS for 3 times and any PBS remaining on the specimen was removed carefully with filter paper. Slides were then incubated with Cy3 fluorescent secondary antibody (1:500) in a humid chamber at room temperature for 1 h, then rinsed 3 times in PBS for 3 min each. DAPI solution was added then the slides were incubated for 2 minutes at room temperature followed by rinsing 3 times in PBS for 3 min each. Anti-fluorescent quencher was added to the tissue sections in the dark. Fluorescence of tissue sections was imaged using a laser scanning confocal microscope.

### Statistical analysis

Randomization was used to assign samples to the experimental groups and treatment conditions for all *in vivo* studies. Data was expressed as mean ± standard deviation (SD). The data was analyzed using analysis of variance (ANOVA) on SPSS/PC* (statistical package for social sciences, personal computer) and Image J software. A value of P<0.05 was considered to be statistically significant.

## Supplementary Material

Supplementary Figures
