# Supplementary material for: Flavonoids from Rosa roxburghii Tratt prevent reactive oxygen species-mediated DNA damage in thymus cells both combined with and without PARP-1 expression after exposure to radiation in vivo
Source: Aging (Albany NY). 2020 Aug 29;12(16):16368–89. doi: 10.18632/aging.103688 (PMC7485694; doi:10.18632/aging.103688)
Supplement: Supplementary Figures [file aging-12-103688-s001..pdf]

SUPPLEMENTARY FIGURES

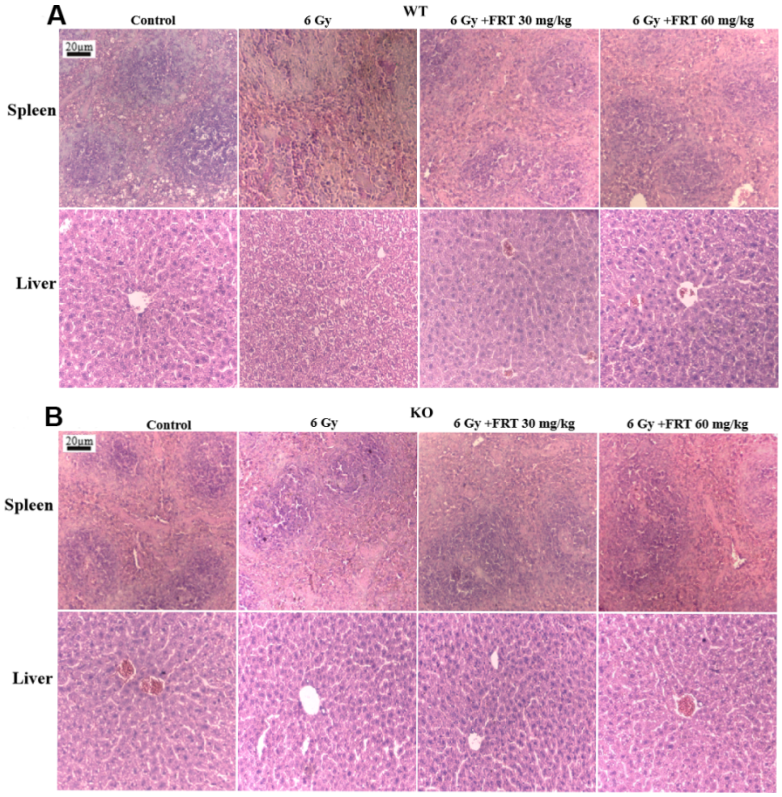

Supplementary Figure 1. Radiation changed the morphology of liver/spleen tissue, but FRT reduced these changes. (**A**) and (**B**). Sacrificed mouse spleens and livers 4 days after radiation were fixed in 4% paraformaldehyde for overnight, embedded in paraffin, sectioned and stained with HE, then imaged using light microscopy.

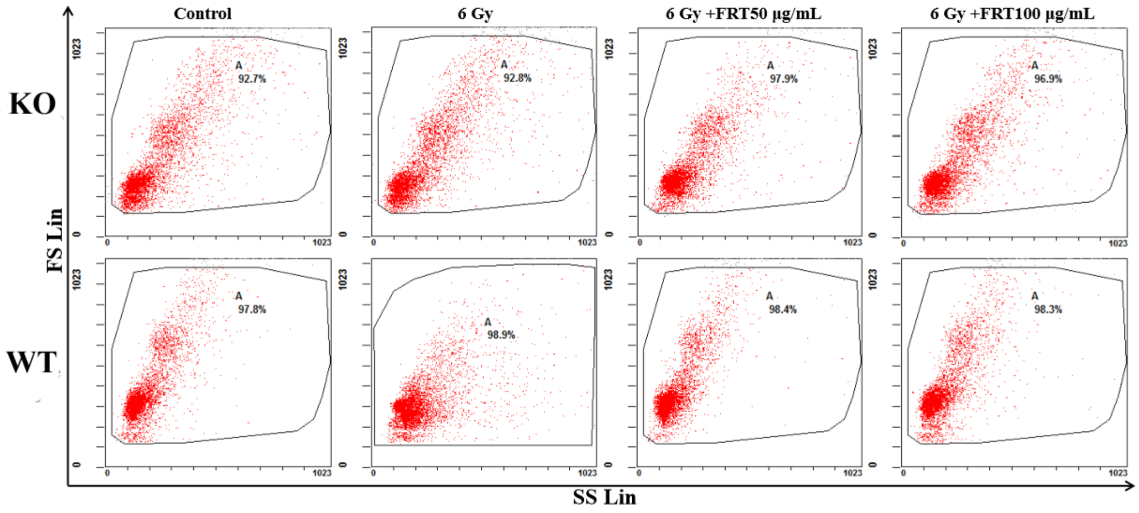

Supplementary Figure 2. Effect of FRT on apoptosis of thymus cells after irradiation due to PARP-1. Apoptosis was detected using an Annexin V-FITC labeled fluorescent probe with green fluorescence. Cells were treated with FRT 2 h before irradiation, then apoptosis was measured 6 h after irradiation.

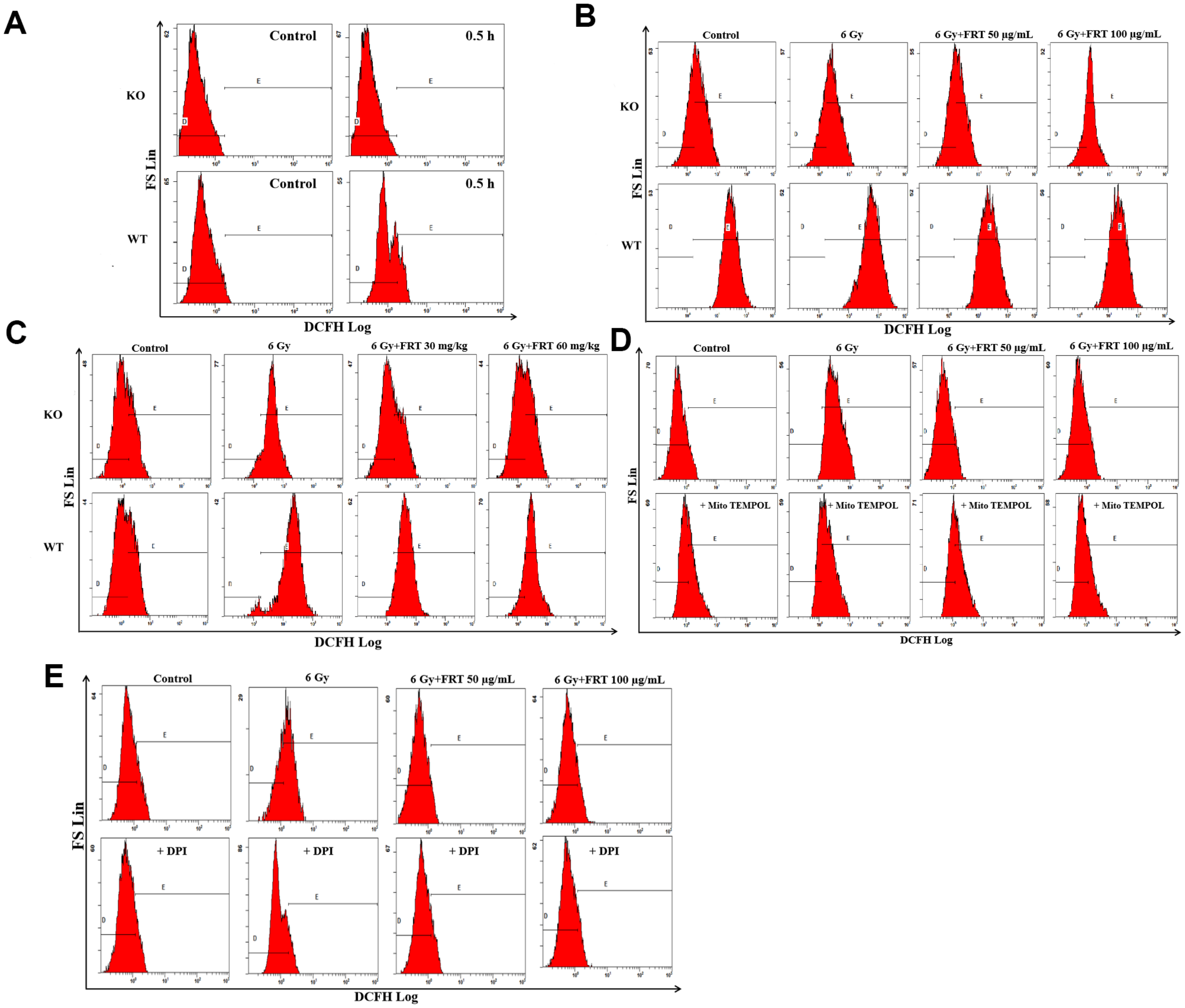

Supplementary Figure 3. Effect of FRT on intracellular ROS scavenging. (**A**) Thymus cell suspensions from WT and KO mice were divided into normal and radiation groups. Thymus cells were harvested 0.5 h after radiation, and levels of reactive oxygen species detected. (**B**). Thymus cell suspensions were divided into four groups: Control, radiation (6 Gy), radiation+FRT 50 μg/mL and radiation+FRT 100 μg/mL groups. After incubation of cells with FRT for 2 hours, thymus cells in the experimental group were given a one-time X-ray radiation of 6 Gy. ROS levels in cells loaded with probes were measured by flow cytometry. (**C**). Thymus cells from WT and KO mice were divided into 4 groups: control, radiation (6 Gy), radiation + FRT 30 mg/kg and radiation + FRT 60 mg/kg groups. Mice in the drug group were administered FRT for 4 days. Mice were exposed to a one-time X-ray radiation dose of 6 Gy. ROS levels in the thymus cells loaded with probes were measured by flow cytometry. (**D**) Thymus cells treated with MitoTEMPOL (50 μM, an anti-oxidant targeting mitochondria) after FRT treatment for 1 h. Cells were exposed to 6 Gy after another 1 h incubation, then ROS levels were detected by flow cytometry 0.5 h after irradiation. (**E**). Thymus cells were treated with DPI (10 μM, an inhibitor of NADPH oxidase) after FRT treatment for 1 h, Cells were exposed to 6 Gy after another 1 h incubation, then ROS levels were detected by flow cytometry 0.5 h after irradiation.

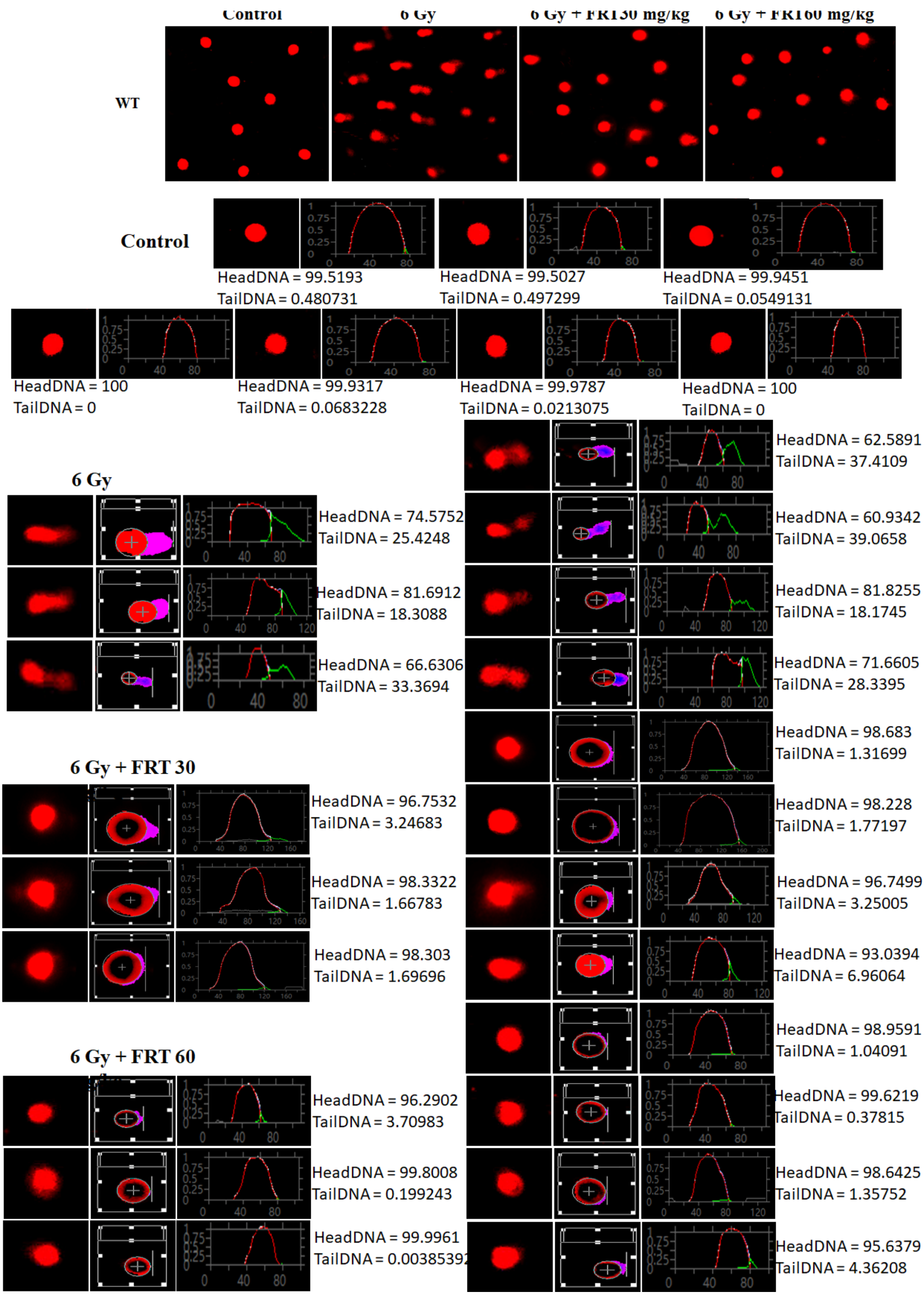

Supplementary Figure 4-1. The effect of FRT on radiation-induced DNA damage measured by CASP software in thymus tissue of WT mice.

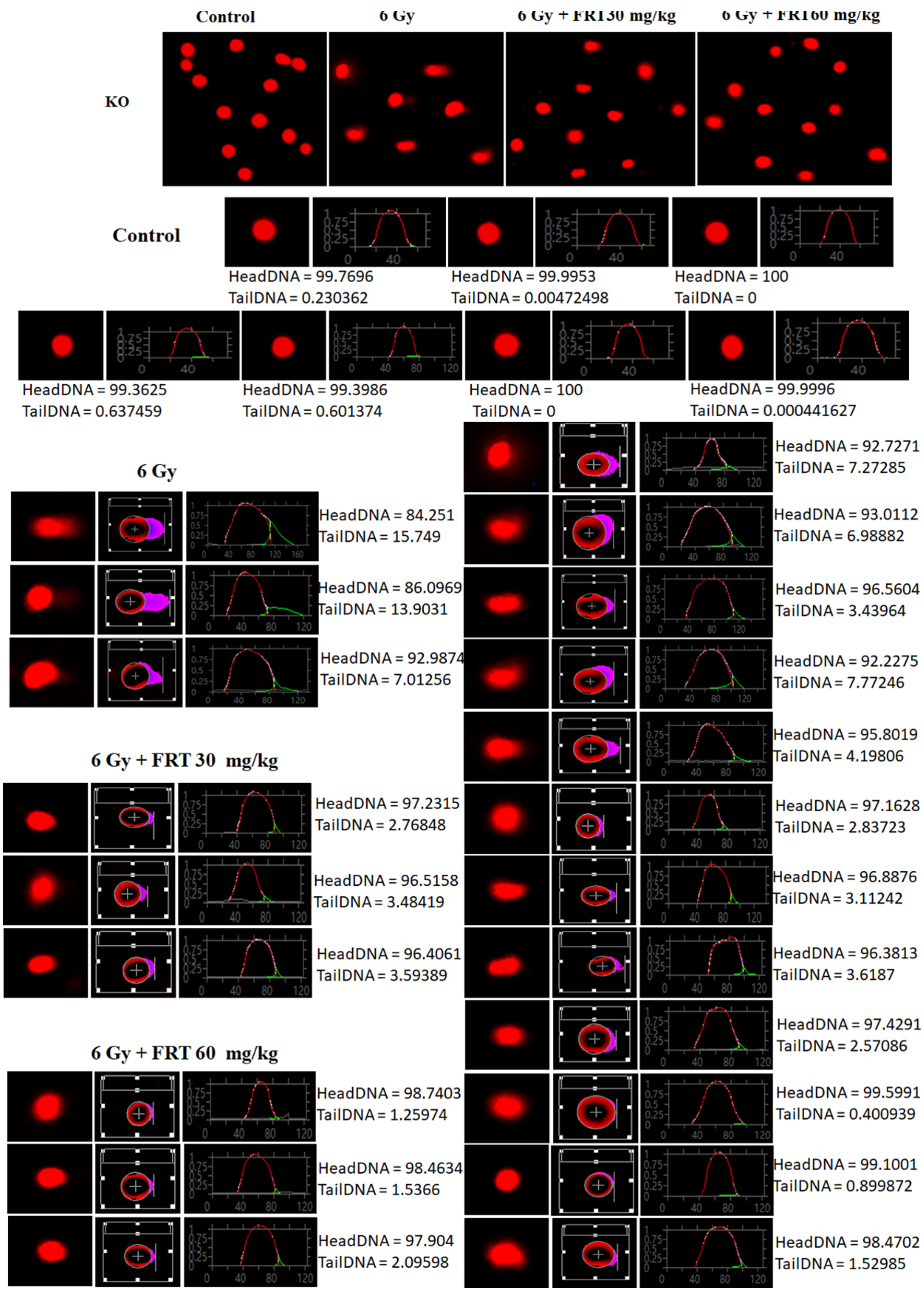

Supplementary Figure 4-2. The effect of FRT on radiation-induced DNA damage measured by CASP software in thymus tissue of KO mice.

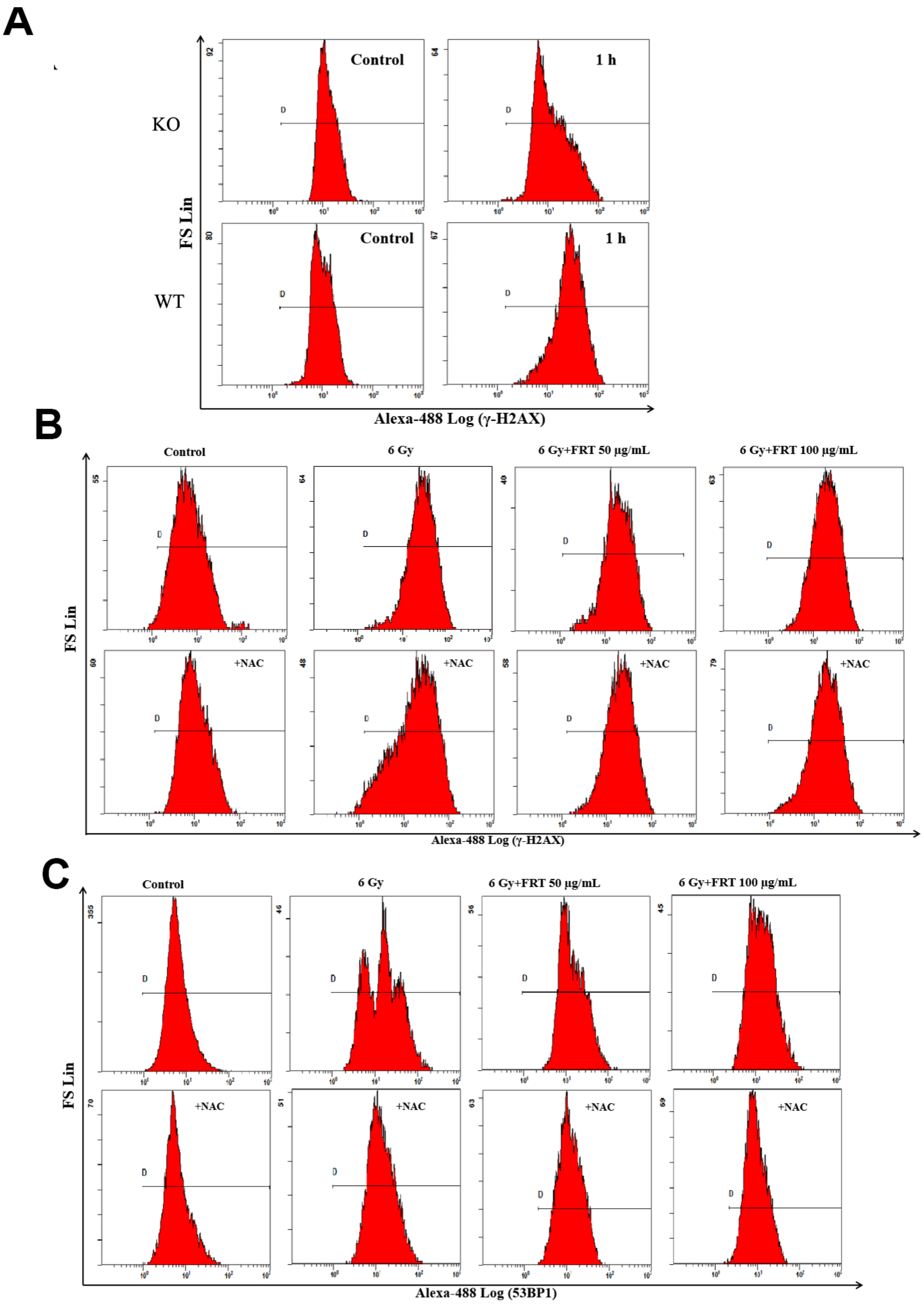

Supplementary Figure 4-3. Protective effect of FRT on radiation-induced thymus DNA damage. (**A**). The thymus from WT and KO mice were harvested and ground into a cell suspension. Intracellular γ-H2AX levels were measured by flow cytometry one hour after 6 Gy irradiation. (**B**) and (**C**). Thymus cells from WT mice were divided into eight groups. [illegible] Blank control group; [illegible] Pure radiation group 6 Gy; [illegible] Radiation group + FRT 50 μg/mL group; [illegible] Radiation group + FRT 100 μg/mL group; [illegible]. Control + NAC (0.5 mM); [illegible] 6 Gy + NAC (0.5 mM); [illegible] 6 Gy + FRT 50 μg/mL+NAC (0.5 mM); [illegible] 6 Gy + FRT 100 μg/mL+NAC (0.5 mM). NAC and FRT were added to cell cultures 2 h before radiation, then collected 1 h after radiation. γ-H2AX and 53BP1 was detected by flow cytometry.
